# Isolation of novel *Lactobacillus* with lipolytic activity from the vinasse and their preliminary potential using as probiotics

**DOI:** 10.1186/s13568-020-01026-2

**Published:** 2020-05-15

**Authors:** Chengran Guan, Zhiqiang Tao, Li Wang, Ruifeng Zhao, Xuan Chen, Xinyuan Huang, Jianbo Su, Zhen Lu, Xia Chen, Ruixia Gu

**Affiliations:** 1 Key Lab of Dairy Biotechnology and Safety Control, College of Food Science and Technology, Yangzhou University, Yangzhou, 225127 Jiangsu China; 2 Bloomage Biotechnology Corporation Limited, Jinan, 250000 Shandong China

**Keywords:** Degrading rate, *Lactobacillus*, Lipase, Lipolytic capacity, Probiotics

## Abstract

*Lactobacillus casei* f1, *L. paracasei* f2 and *L. paracasei* f3 with lipolytic activity were isolated and identified from vinasses according to the morphological–physiological properties detection and 16S rDNA analysis. These three strains showed obvious lipase activities to olive oil and *L. casei* f1 performed highest enzyme activity of 17.8 U/mL. *L. casei* f1, *L. paracasei* f2 and *L. paracasei* f3 could lipolyze the blending oils, peanut oil and sesame oil with diverse degrading rates. The degrading rates to the preferred oils, *L. casei* f1 to blending oils, *L. paracasei* f2 to peanut oil and *L. paracasei* f3 to sesame oil, were 21.2%, 27.3% and 39.6%, respectively. The corresponding oil degrading rates increased as the cell growth and the highest degrading rates were obtained at the stationary phase with the viable count more than 7.5 LogCFU/mL. By GC–MS analysis, *L. casei* f1, *L. paracasei* f2 and *L. paracasei* f3 performed diverse lipolytic capacities to the 12 kinds of fat acids and all of them preferred to hydrolyze the linoleic acid with the degrading rate of 49.11%, 31.83% and 64.44%, respectively. These three strains showed considerable probiotic properties, displaying higher than 10^6^ CFU/mL desirable viable count though the simulated gastrointestinal tract, as well as inhibiting six indicator bacteria. These results suggested that the three isolated strains could be considered as novel probiotic candidates and applied in the food industry.

## Introduction

Lipases (triacylglycerol acylhydrolases EC: 3.1.1.3) are ubiquitous enzymes which catalyze the hydrolysis of triacylglycerols to give free fatty acids, diacylglycerols, monoacylglycerols and glycerol (Liu et al. 2006). The considerable physiological significance and industrial potential characteristics of lipases make them wildly applied in a large number of hydrolytic applications, like flavor development in dairy products (cheese, butter and margarine), alcoholic beverages, milk chocolate, etc. (Sharma et al. 2001; Konkit and Kim 2016). Other than the food industry, lipases could also be used for synthesis of ingredients for personal care products, the synthesis of surfactants, the pesticide industry and the environmental management (Esteban-Torres et al. 2015; Haghshenas et al. 2017).

Lipase were reported to be produced by plants, animals, and microbes. Most of the industrial lipase were derived from fungi and bacteria, such as *Aspergillus* sp., *Bacillus* sp. and *Pseudomonas* sp. (Fleuri et al. 2014; Rathi et al. 2016; Hu et al. 2018). Microbial lipases are of special interest owing to their stability in organic solvents, lack of requirement for cofactors, broad substrate specification and high enantioselectivity (Gupta et al. 2004). It is demonstrated that the lipase originated from different strains is specific in the exquisite chemoselectivity, regioselectivity and stereoselectivity (Magadum and Yadav 2018).

A large number of methods had been developed to detect lipase producing microorganisms (Deeth and Touch 2000). One of the most employed strategy is using the medium containing special substrate to isolate the microorganisms with lipase activity. The frequently used substrates were olive oil, sunflower seed oil, soya oil, salad oil, fatty acids containing even or odd numbered carbon atoms and so on (Brooksbank et al. 2007; Matsumiya et al. 2007). The lipolytic activity to the substrate was demonstrated to be strain dependent. *Lactobacillus casei* L-7, *L. casei* L-14, *L. plantarum* L-34 and *L. helveticus* L-7 were reported to be lipolytic and all of the four strains could hydrolyze the milk fat and olive oil with the exception of *L. casei* L-7, which failed to hydrolyze olive oil (Brooksbank et al. 2007). The production of lipase was mostly inducer dependent, and in many cases vegetable oils act as good inducers on account of the presence of higher percentage of long chain fatty acid esters, availability and low costs (Lakshmi et al. 1999).

Lactic acid bacteria (LAB) are attractive for their status of generally recognized as safe and attractive probiotic characteristics. LAB with lipolytic capacity were identified to be unique to hydrolyze a range of fatty acids, tri, di, and monoacylglyceride substrates which are important to food industry, especially the dairy and meat industry (Aravindan et al. 2007). Although there were reports on lipase production from LAB, they were generally considered to be weakly lipolytic, as compared to other groups of microorganisms (Esteban-Torres et al. 2015). Moreover, there were only a small part of LAB genera possessing lipolytic activity. Meyers only isolated 29 lipase-producing strains from over 100 different LAB representing the genera of *Lactobacillus*, *Lactococcus*, *Leuconostoc*, *Pediococcus* and *Streptococcus* (Meyers et al. 1996). In brief, due to the generally low lipase activity, substrate specific and limited resources, the quantity of LAB with lipolytic activity were limited.

To cater to the specific industrial needs, novel LAB with desired lipolytic properties have to be isolated. In this work, LAB with lipolytic activity were isolated and identified from vinasse and the lipolytic related properties and probiotic characteristics were studied. The results suggested that the isolated strains could be wildly applied in food industry.

## Materials and methods

### Raw material and mediums

Vinasse samples were obtained from Jin Danyang Wine Winery (Danyang, Jiangsu, China). With the help of a sterile spatula, five samples were obtained from different mounds of vinasses and stored in sterile plastic bags. Then, the vinasse sample was transported to the laboratory under sterile conditions. The oils and mediums used in this work were listed in Table 1.

**Table 1:** Oils and mediums used in this study

Oils and mediums	Characteristics
Oils	
Blending oils	8 kinds of vegetables oils (soybean oil, rapeseed oil, sunflower seed oil, peanut oil, rice oil, corn oil, sesame oil, flaxseed oil), sold by Yihai Kerry Food Marketing Co., Ltd.
Peanut oil	Pressed from peanuts with proprietary technology, sold by Shandong Luhua group Co., Ltd.
Sesame oil	pressed from sesame with special procession, sold by Shanghai Taitaile Food Co., Ltd.
Mediums	
Basal medium	MRS medium
Mineral medium	Basal medium without yeast extract, beef extract, peptone extract and phytone
Screening medium	Glucose was replaced by 20 g/L blending oils in MRS medium
B-screening medium	Mineral medium with 20 g/L blending oils
P-screening medium	Mineral medium with 20 g/L peanut oil
S-screening medium	Mineral medium with 20 g/L sesame oil

### Strain isolation

Thirty grams of the vinasse sample was homogenized in 270 mL 0.8% sterile saline solution and spread on the MRS medium, and then separately placed in the 37 °C and 42 °C constant temperature incubator for 48 h. The colony which were morphologically like the lactic acid bacteria were isolated. Moreover, the obtained colony were further sequentially screened on the screening medium and B-screening medium and the Gram-positive strains with the morphology like lactic acid bacteria were isolated.

### Physiological and biochemical identification

The strains isolated from B-screening medium were executed physiological and biochemical tests according to the Berger Bacterial Identification Manual, including catalase assay, sugar (glucose, sucrose, lactose, maltose) fermentation test, starch hydrolysis test, Gelatin hydrolysis test, methyl red test, nitrate reduction test, hydrazine test and hydrogen sulfide test.

## 16S rDNA sequence analysis

The chromosome DNA of the isolated strain was firstly extracted using an Ezup column bacterial genomic DNA extraction kit (Sangon, Shanghai). Using primer 16S-27F (5′-AGAGTTTGATCCTGGCTCAG-3′) and primer 16S-1492R (5′-TACGGCTACCTTGTTACGACTT-3′), the 16S rDNA was amplified from the corresponding chromosome DNA. After purification by electrophoresis, the product was sent to be sequenced (Bioengineering Co., Ltd, Shanghai). The analysis of the 16S rDNA sequence was carried out by the NCBI online blast. Then, the 16S rDNA sequences of *L. casei* f1, *L. paracasei* f2 and *L. paracasei* f3 have been deposited in GenBank with the submission number of MN744421, MN744423, MN744424, respectively.

### Detection of lipase activity

Four conical flasks numbered as 0, 1, 2 and 3 were prepared and 15 mL of 95% alcohol was added into flask 0, while 5 mL of the substrate and 5 mL of phosphate buffer (pH 4.8, 20 mmol/L) were added into flasks 1, 2 and 3. The substrate was prepared by adding 50 mL olive oil into 150 mL polyvinyl alcohol (PVA, 2%) and blending on the vortex mixer for 3 min. One milliliter of *L. casei* f1, *L. paracasei* f2 and *L. paracasei* f3 cultivating in MRS medium for 24 h were separately added into flasks 1, 2 and 3 and mixed quickly. The reaction was carried out and put at 30 °C for 10 min, after which the enzyme activity was stopped by adding 15 mL of 95% ethanol. The liberated free fatty acid was titrated against 0.1 M NaOH using phenolphthalein as the indicator. One unit of lipase is defined as the amount of enzyme, which liberates 1 μmol fatty acid.

### Determination the degrading rate of the oil

The target strain was separately incubated in the B-screening medium, P-screening medium and S-screening medium. After cultivation at 37 °C for 48 h, the supernatant was harvested by centrifugation, extracted by 30 mL of n-hexane for three times and the organic layer was collected in the reservoir bottle. Furthermore, the bottle was transformed to the rotary evaporator to get rid of the residual organic solvent of the sample. After that, the sample was placed at 60 °C for 1 h and then cooled in the drier for 0.5 h and then be weighed. Repeat the above procedure until the weight was constant (the difference between two continuous weight was within 1 mg). The oil degradation rate was calculated as follows:

$${\text{X }} = [ 1- ({\text{m2}} - {\text{m1}}) \, /{\text{ m}}0] \times { 1}00\%$$

In the formula:

X-the oil degradation rate (%); m0-the amount of the oil added in the medium, in gram (g); m1-the weight of the reservoir bottle, in gram (g); m2-the weight of the reservoir bottle containing the dried sample, in gram (g).

### Detection of oil degrading curve

*Lactobacillus casei* f1, *L. paracasei* f2 and *L. paracasei* f3 were separately inoculated (3% v/v) into 10 mL of B-screening medium, P-screening medium and S-screening medium and then were cultivated at 37 °C for 48 h. The sampling was implemented throughout the process with an interval of 4 h. Each sample was used to detect the oil degradation rate by the previous paragraph and the viable count was performed at the same time (Soomro and Masud 2012).

### Analysis of the degraded specific fatty acids by GC–MS

Qualitative and quantitative analysis of fatty acids was performed as previously reported with slight modification (Sharma et al. 2001). Briefly, *L. casei* f1, *L. paracasei* f2 and *L. paracasei* f3 were inoculated (3% v/v) into 10 mL of B-screening medium, P-screening medium and S-screening medium, respectively. After cultivating for 48 h, NaOH-methyl alcohol (5.0 mL 0.5 mol/L) was dissolved into the medium to perform methyl esterification at 50 °C for 30 min. After cooling for 2 h at room temperature, five milliliters of hexane was added and vortexed for 30 s. After phase separation, the superior organic-phase was injected (1 µL, splitless mode) in the Trace DSQII gas chromatograph (Thermo Finnigan, San Jose, CA, USA) with a capillary column (30 m × 0.25 mmID × 0.25 μm, Agilent, Agilent Technologies, Waldbronn, Germany). The Mass spectrum splitting voltage was 70 eV and the carrier gas flow (helium) was 1 mL/min. The chromatogram was recorded in the scan mode (30–450 m/z) with a programmed temperature (50 °C for 20 min, warming up to 200 °C by 4 °C/min and then keeping for 5 min, warming up to 220 °C by 4 °C per min and then keeping for 20 min). The fatty acid species were determined based on the retention time of the standard and mass spectrometry cleavage information, and the fatty acid content per milliliter of bacterial suspension was expressed as an equivalent mass of fatty acid methyl ester.

### In vitro determination of probiotic properties

Acid and bile salt tolerance assay: The tolerance assay was processed as previously reported with modifications (Haghshenas et al. 2017). The strain was inoculated into 10 mL of MRS medium cultivating at 37 °C for 24 h. The culture was centrifuged at 10,000 rpm/min for 10 min to obtain the pellet. The pellet was washed twice and suspended in the 0.85% (w/v) physiological saline. One milliliter of strain suspension was separately mixed with 9 ml of the simulating gastric juice (pH at 2.0 and 3.0), intestinal fluid and bile salt medium (1% and 3%) for 3 h. The viable count was performed and the survival rate was calculated as the percentage of Log colonies grown compared to the initial concentration (Soomro and Masud 2012).

Extracellular antimicrobial activity assay: The antimicrobial activity of the supernatants from the isolates were determined with slight modification as previously reported (Bauer et al. 1966). The tested strain was cultivated in the tube containing 5 mL of MRS medium for 24 h and then centrifugated to collect the supernatants for antimicrobial activity assay. The colony of the indicator bacteria (*Listeria monocytogenes*, *Staphylococcus aureus* CICC21600, *E. coli* EPEC CICC10664, *Salmonella* CICC21513, *Bacillus cereus* CICC21261, *B. subtilis*) was inoculated into a tube containing 5 mL of LB medium and cultivated at 37 °C for 12 h. One milliliter of the bacterial suspension diluting to 10^6^ CFU/mL was used to spread on the pre-dried plate. After the inoculum drying, three holes per plate were processed by a puncher with 6 mm diameter. Two hundred microliter of the supernatants of the tested strain were added into the hole spreading at 4 °C for 4 h. And then, the plate was cultivated at 37 °C for 8 h and the zone diameters (including the 6-mm hole) were measured with a ruler.

All of the experiments were carried out with triplicate parallel.

### Data analysis

Data analysis was done by SPSS 20.0 software (SPSS Inc., Chicago). All the experiments were repeated three times unless stated otherwise. The statistical analysis was carried out using one-way analysis of variance (ANOVA).

## Results

### Isolation of lipid-degrading lactic acid bacteria

**Fig. 1:**
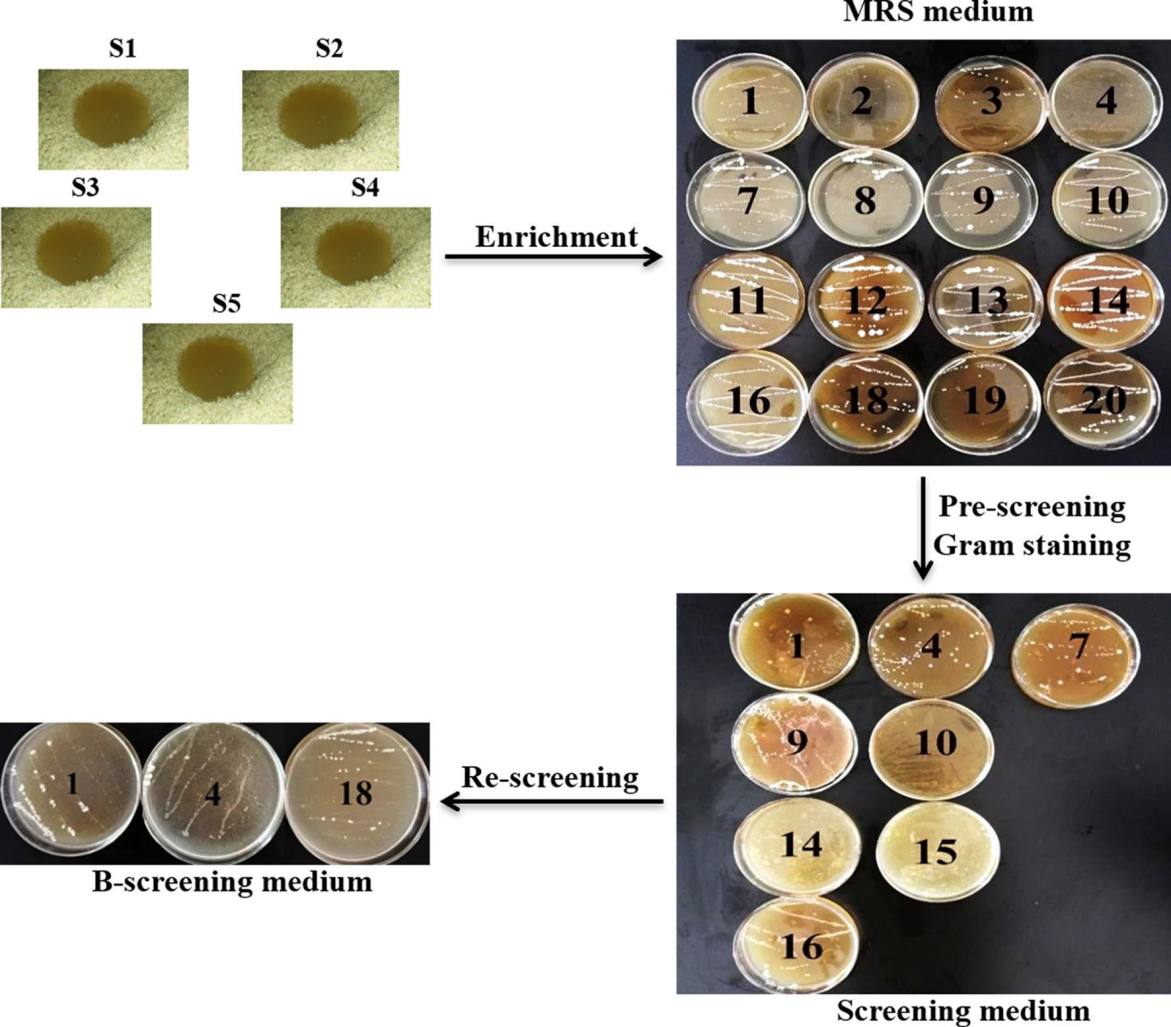
Flow chart for the isolation of LAB with lipolytic capacity. Vinasses were sampled from the wine factory. After enrichment on the MRS medium, 16 strains with LAB-like morphology were firstly selected. Using screening medium for prescreening, 8 strains were isolated which could grow on the medium as well as Gram-positive. Moreover, strain 1, 4, 18 were successfully re-screened with the B-rescreening medium

Twenty colonies (named 1 to 20 in sequence) were firstly obtained from the collected samples by enrichment on the MRS solid medium (Fig. 1 and Additional file 1: Table S1). By observing the growth-related characteristics of each colony, colonies 5, 6, 15 and 17 were abandoned owing to their mold-like morphology. The other 16 colonies were morphological like the lactic acid bacteria and were chosen to be pre-screened on the screening medium (Fig. 1). Only 8 strains (1, 4, 7, 9, 10, 14, 15 and 16) could grow on the screening medium and further demonstrated to be positive by Gam staining analysis (Additional file 1: Table S2). Then, to eliminate the influence of the organic carbon and nitrogen source, the 8 strains were re-screened on the B-rescreening medium. Finally, only strain 1, 4 and 18 were isolated (Additional file 1: Table S3).

### Identification of lipid-degrading lactic acid bacteria

**Fig. 2:**
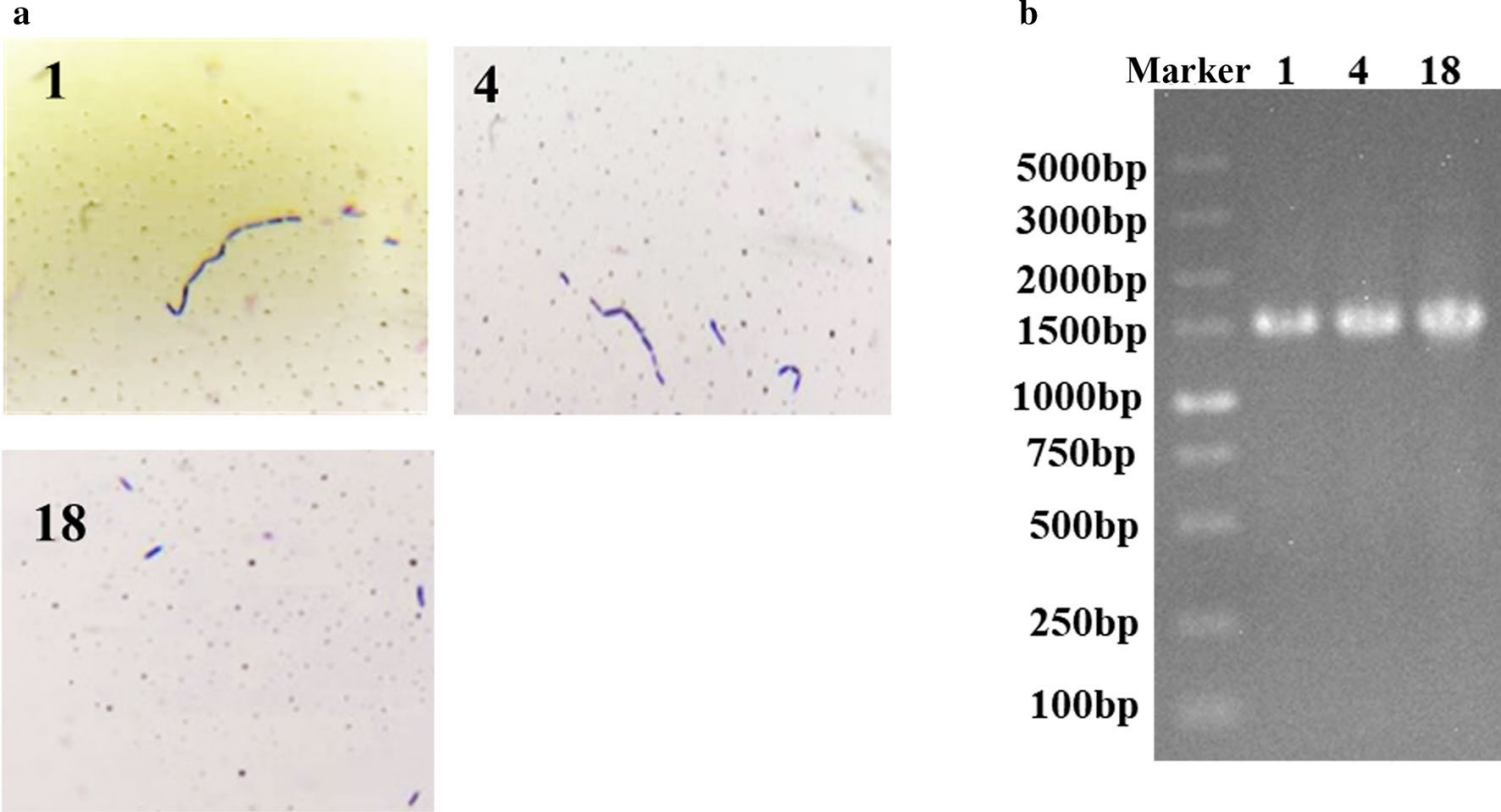
The morphology and the 16S rDNA analysis of the isolated strains. The isolated strains were dyed and the morphology were observed (**a**). The amplified 16 s rDNA was analyzed by nucleic acid gel (**b**)

The physiological and biochemical characteristics of the strain 1, 4 and 18 were determined (Table 2 and Fig. 2a). The physicochemical indexes of the three Gram-stain-positive strains were in accordance with the description for lactic acid bacteria in Berger’s bacterial identification manual. Moreover, the 16S rDNA of the three strains were separately amplified and sequenced (Fig. 2b, Additional file 1: Table S4). By blasting with the deposited sequences in GenBank, strain 1 possessed a 16S rDNA sequence with 99% similarity to *Lactobacillus casei* while the 16S rDNA sequence of strain 4 and 18 showed 99% similarity to *L. paracasei*. Combining with the biochemical, morphological, and physiological properties, strain 1, 4, 18 were correspondingly renamed as *L. casei* f1, *L. paracasei* f2 and *L. paracasei* f3 and then stored in the China general microbiological culture collection center with the corresponding No. 18353, 18354 and 18355.

**Table 2:** The biological characteristics of the strains

Tests	Response		
	1	4	18
Hydrogen peroxide test	−	−	−
Starch hydrolysis	−	−	−
Methyl red test	+	+	+
Gelatin liquefaction	−	−	−
Acid production from glucose	+	+	+
Gas production from glucose	−	−	−
Nitrate reduction	−	−	−
H_2_S production	−	−	−
Mobility	−	−	−

“+” is positive and “−” is negative

### Lipase activity

**Fig. 3:**
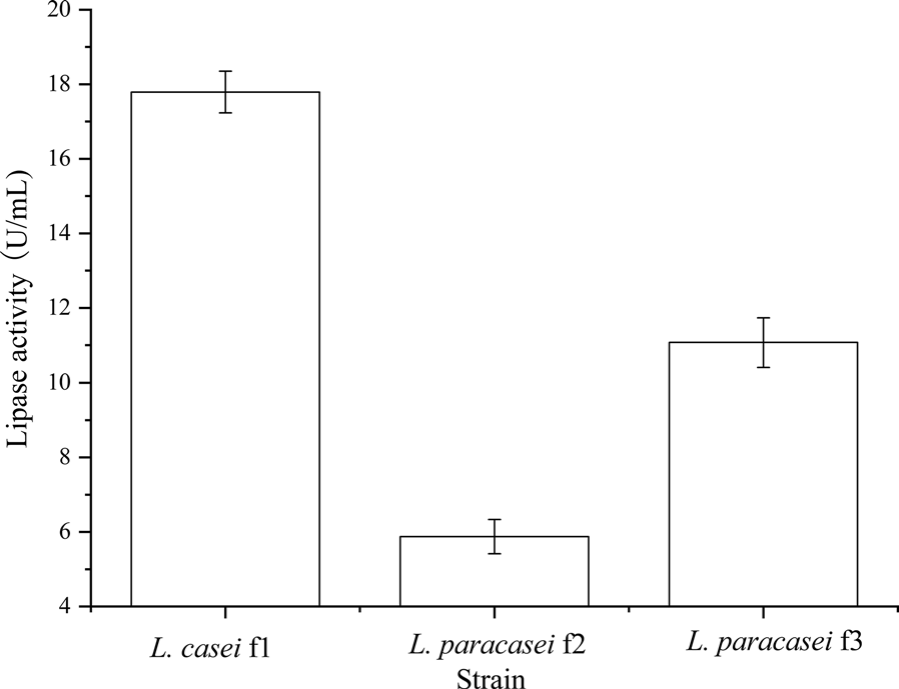
Lipase activity of the isolated strains. Using the olive oil as the substrate, the lipase activity of *L. casei* f1, *L. paracasei* f2 and *L. paracasei* f3 were separately detected. Data are presented as mean ± SD (n = 3). The vertical bar represents standard errors of mean

In this work, using olive oil as the substrate, the lipolytic capacity of *L. casei* f1, *L. paracasei* f2 and *L. paracasei* f3 were evaluated by detecting the lipase activity (Fig. 3). The lipase activities of the three strains varied in the range from 5.88 to 17.79 U/mL. The highest lipase activity was detected in *L. casei* f1 which was 2.02 and 0.6 times higher than that of *L. paracasei* f2 and *L. paracasei* f3, respectively.

### Lipid degradation

**Fig. 4:**
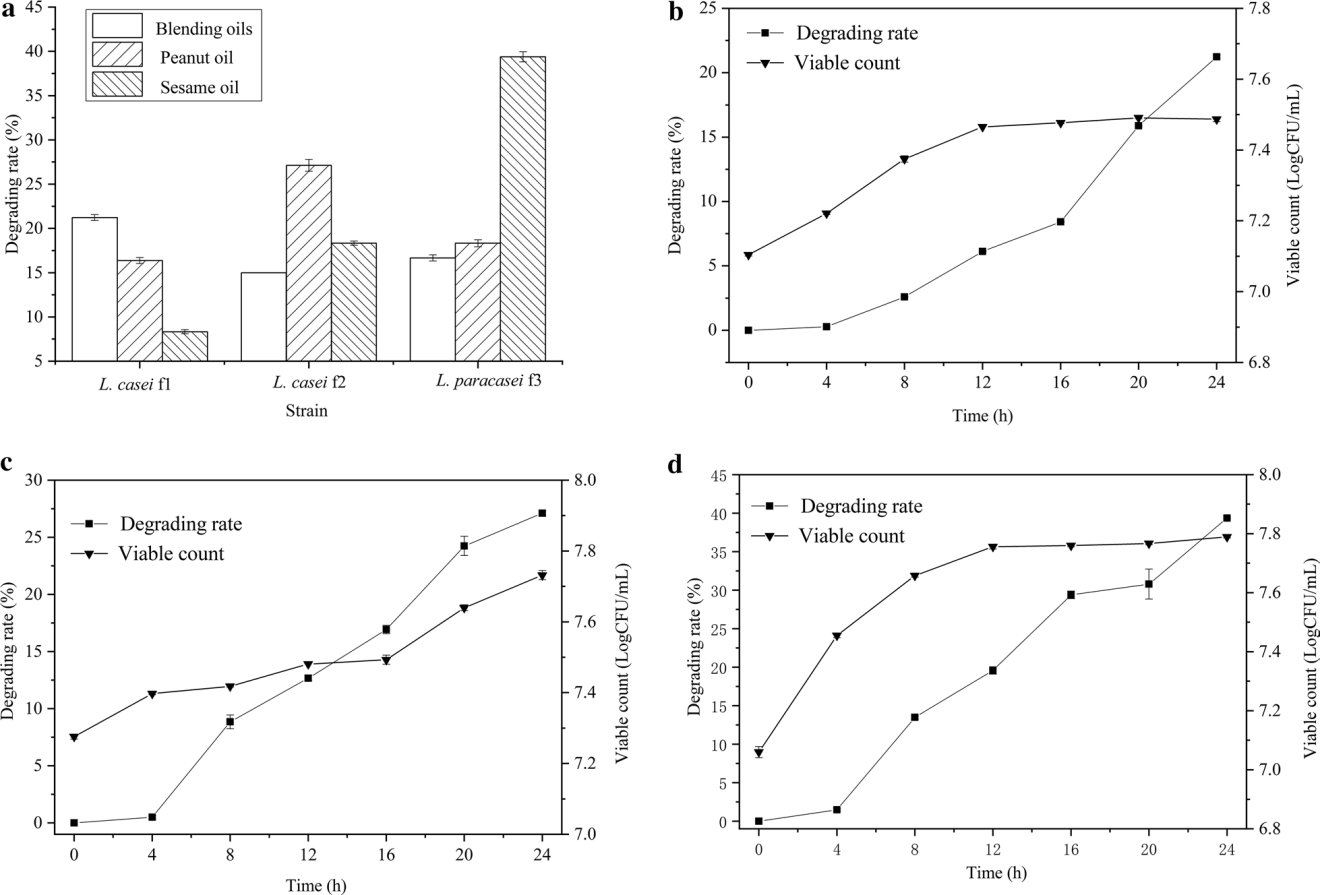
The oil degrading activity of the isolated strains. *L. casei* f1, *L. paracasei* f2 and *L. paracasei* f3 were separately cultivated for 48 h in the mineral mediums containing blending oils, peanut oil and sesame oil as the sole carbon source, respectively. The weight of the corresponding degraded oils was measured (**a**). Moreover, sampling was implemented with an interval throughout the process when the strains cultivated in the mineral medium containing the preferred oils. The corresponding cell growth and the degraded oils of *L. casei* f1 to blending oils (**b**), *L. paracasei* f2 to peanut oil (**c**) and *L. paracasei* f3 to sesame oil (**d**) were analyzed. Data are presented as mean ± SD (n = 3). The vertical bar represents standard errors of mean

To test the oil-specific characteristics of the isolated strains, *L. casei* f1, *L. paracasei* f2 and *L. paracasei* f3 were separately cultivated in the medium containing blending oils, peanut oil or sesame oil as the solo source of carbon. The oil degrading rate were determined by constant weight method. All of the three strains could degrade the target oils with diverse degrading rates (Fig. 4a). *L. casei* f1, *L. paracasei* f2 and *L. paracasei* f3 separately tended to degrade blending oils, peanut oil and sesame oil.

Moreover, the degrading curves of *L. casei* f1 to blending oils, *L. paracasei* f2 to peanut oil and *L. paracasei* f3 to sesame oil were measured (Fig. 4b–d). All of these three strains showed very good growth in the inorganic medium just containing the corresponding oil as the solo carbon source. The bacteria entered into stationary phase after 12 h and the viable count could reach to more than 7.5 LogCFU/mL. Meanwhile, the degrading rate of the oil persistently increased from the late log phase to the stationary phase. After cultivation for 24 h, the degrading rate of *L. casei* f1 to blending oils, *L. paracasei* f2 to peanut oil and *L. paracasei* f3 to sesame oil could be 21.18%, 27.30% and 39.63%, respectively.

### Fatty acids analysis

**Fig. 5:**
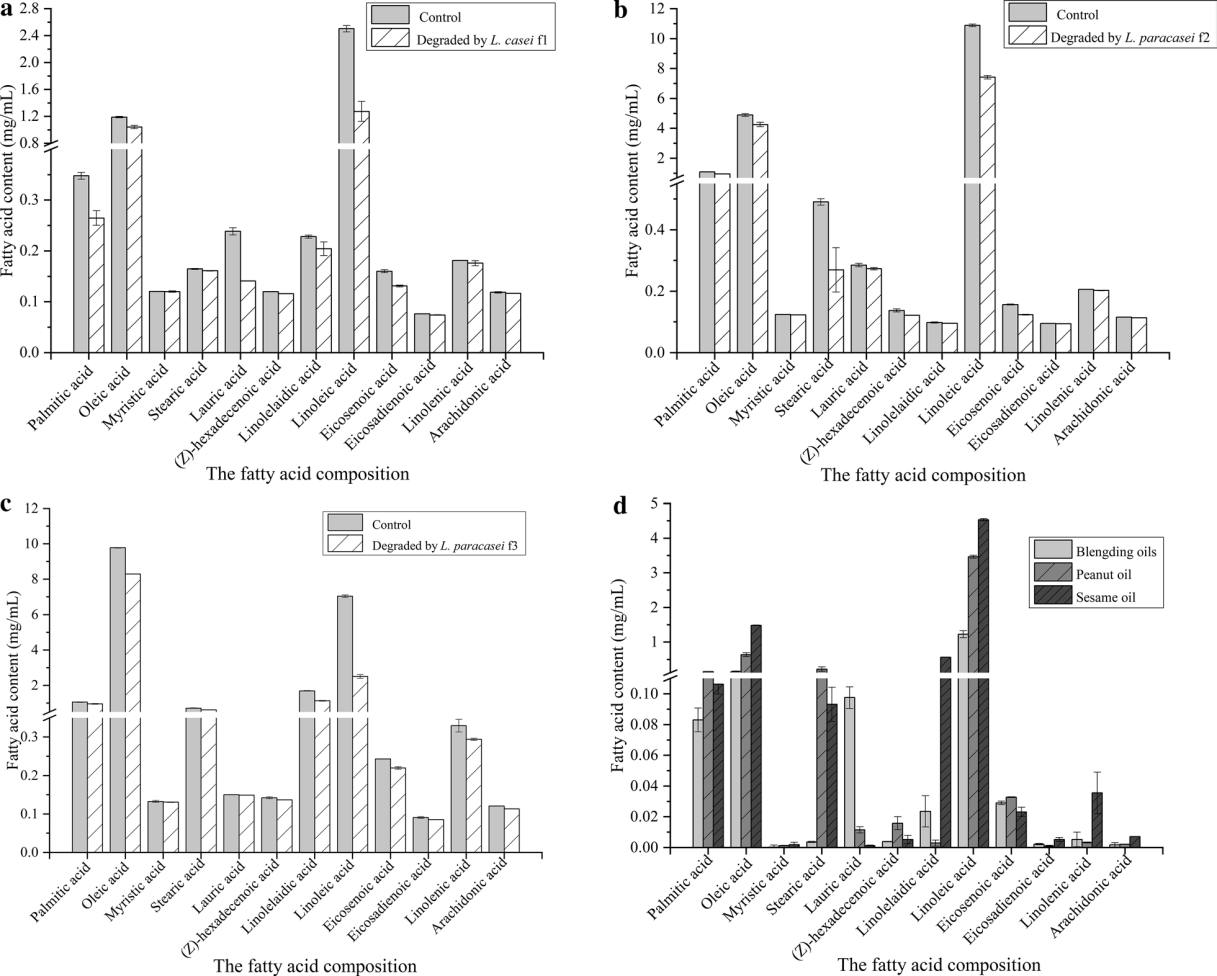
The fat acids of the preferred oils degraded by the isolated strains. To detect the degraded fat acids of the oil, *L. casei* f1 (**a**), *L. paracasei* f2 (**b**) and *L. paracasei* f3 (**c**) were separately cultivated for 48 h in the mineral medium containing the blending oils, peanut oil and sesame oil as the solo carbon source, respectively. The variation of the fat acids in each sample was analyzed (**d**). Data are presented as mean ± SD (n = 3). The vertical bar represents standard errors of mean

Furthermore, the composition of the corresponding degraded oil was analyzed by GC–MS (Fig. 5). There were only 12 kinds of fatty acids could be detected in this study. The hydrolyze capacities of *L. casei* f1 to blending oils, *L. paracasei* f2 to peanut oil and *L. paracasei* f3 to sesame oil were separately 1.63 mg/mL, 4.54 mg/mL and 6.86 mg/mL. The main fatty acids in these three kinds of oils were linoleic acid and oleic acid. After degradation, the oleic acid and linoleic acid were highly hydrolyzed. *L. casei* f1, *L. paracasei* f2 and *L. paracasei* f3 were all tend to hydrolyze the linoleic acid with the hydrolyzed rate of 49.11%, 31.83% and 64.44%, respectively (Fig. 5a–c). In addition, the fatty acids of low content were also slightly degraded (Fig. 5d).

### Properties considered important for probiotic function

To determine the specific survival percentage after passage through the human gastrointestinal tract, the survival rates of the three isolated strains were detected after 3 h of incubation in the simulating gastric juice, intestinal fluid and bile salt (Table 3). *L. casei* f1, *L. paracasei* f2 and *L. paracasei* f3 were demonstrated a low capacity to withstand exposure to the simulated pH conditions. Compared with *L. casei* f1 and *L. paracasei* f2, *L. paracasei* f3 showed higher tolerance to acid at pH 3.0. However, the survival rate of *L. casei* f1 reduced significantly at pH 2.0. In addition, all the tested strains showed weak tolerance to bile salt as the survival rates dramatically reduced with the increased concentration of bile salt. Moreover, the tested strains performed high survival in the artificial intestinal juice.

**Table 3:** The tolerant capacity of the strains to the simulated gastrointestinal environment

Strain	Control (10^8^ CFU/mL)	Survival rate (%)				
		Artificial gastric juice		Bile salt		Artificial intestinal juice
		pH 3.0	pH 2.0	0.1%	0.3%	
*L. casei* f1	4.25 ± 0.45	7.88 ± 1.29	0.37 ± 0.012	9.76 ± 0.12	0.068 ± 0.007	32.8 ± 3.41
*L. paracasei* f2	5.0 ± 0.2	10.7 ± 0.1	3.7 ± 0.1	4.42 ± 0.12	0.055 ± 0.013	7.2 ± 1.4
*L. paracasei* f3	6.05 ± 0.15	17.11 ± 0.41	0.034 ± 0.003	12.4 ± 1.82	0.058 ± 0.02	18.26 ± 0.91

**Fig. 6:**
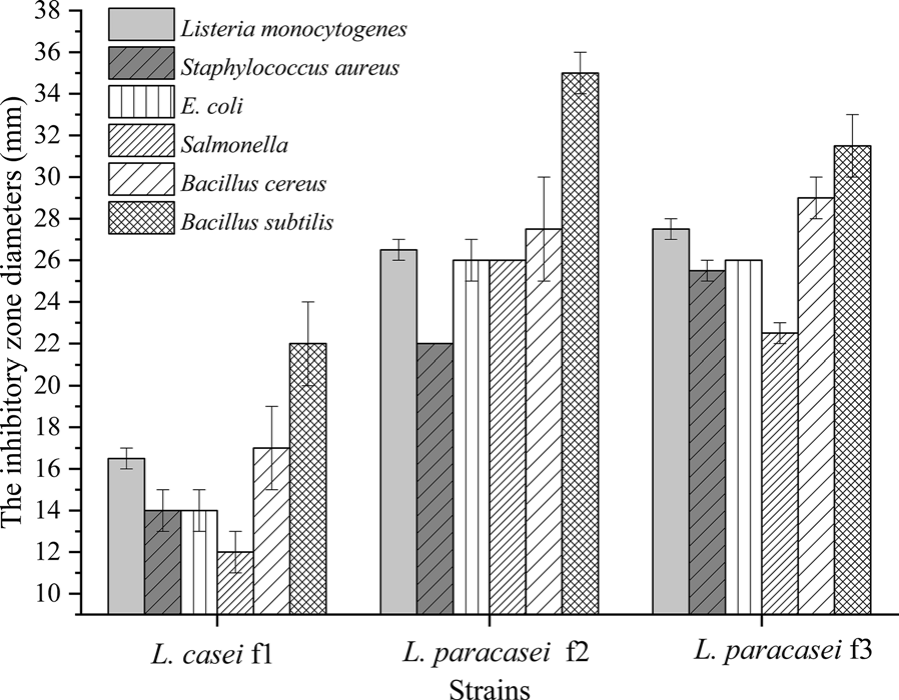
The antimicrobial activity of *L. casei* f1, *L. paracasei* f2 and *L. paracasei* f3. Six common intestinal pathogens were used as indicator bacteria and the diameter of inhibition zones were measured. Data are presented as mean ± SD (n = 3). The vertical bar represents standard errors of mean

To determine the capacity of inhibiting the growth of the pathogenic bacteria, the inhibitory activity of the tested strain was ranked according to the size of zones of inhibition against common intestinal pathogens (Fig. 6). Six indicator bacteria were inhibited by the supernatants of *L. casei* f1, *L. paracasei* f2 and *L. paracasei* f3. There was no particular trait as to the antagonism to Gram-positive and negative indicator bacteria. Moreover, the inhibition zones *L. paracasei* f2 and *L. paracasei* f3 were prominently larger than that of *L. casei* f1.

## Discussion

The microorganisms which could industrially produce lipase were most of fungi and bacteria like *Bacillus* spp., *Candida* spp., *Pseudomonas* spp., while LAB was demonstrated to be weak lipolytic and limited numbers of LAB were identify to be able to degrade lipid. However, LAB with lipolytic capacity were identified to be unique to hydrolyze a range of fatty acids substrates which were important to food industry, especially the dairy and meat industry. As LAB with lipolytic activity are usually isolated from dairy and fish waste, it is necessary to find different sample resources to isolate novel LAB with distinctive capacity of degrading lipid. Baijiu, known as the national liquor of China, is one of the six famous distilled spirits in the world which is usually produced by several processes, including milling of the grains, cooking, mixing with water and Daqu powder, fermentation, distillation with steam and then aging for several years to develop the bouquet aroma (Li et al. 2008). The vinasses is the surplus of Baijiu rich in proteins, amino acids, lipids, carbohydrates, vitamins, trace elements and other nutrients (Espana-Gamboa et al. 2011; Li et al. 2013). Besides the vast nutrients, the vinasses is special for its specific flavor of bouquet suggesting the existence of lipase. In this work, *L. casei* f1, *L. paracasei* f2 and *L. paracasei* f3 with lipolytic capacity were isolated from the vinasses.

The lipolytic capacity of the LAB could be identified by detecting the activity of the lipase (Deeth and Touch 2000). The lipase production is demonstrated to be organism specific and mostly substrates dependent. In many cases, oils act as good inducers of the lipase production (Yu et al. 2003). Among various oils, the olive oil containing high content of oleic acid was demonstrated to be the optimal substrate for the lipase production from *Rhizopus chinensis* (Wang et al. 2008). Using the olive oil as the substrate, *L. casei* f1, *L. paracasei* f2 and *L. paracasei* f3 showed obvious lipase activity and *L. casei* f1 performed highest enzyme activity of 17.8 U/mL. Moreover, the lipase activity was reported to be closely related to the substrate, medium and the cultivated conditions (Liu et al. 2006). The lipolytic activity of lipase from the *Lactobacillus* sp. G5 was reported to be greatly varied with different substrates and the highest enzyme activity of 31.27 U/mL was obtained by using p-nitrophenyl acetate as the substrate (Rashmi and Gayathri 2014). *Enterococcus faecium* MTCC 5695 and *Pediococcus acidilactici* MTCC 11361 showed 3.15- and 2.3-fold increase in lipase production by optimizing the medium components and cultural conditions, respectively, as compared to unoptimized conditions (Ramakrishnan et al. 2013). And the increase in lipase production under optimized conditions was also observed in *Arthrobacter* sp. BGCC#490, *Enterobacter aerogenes* and *Burkholderia* sp. (Liu et al. 2006; Kumari et al. 2009; Sharma et al. 2009). Therefore, the lipase activities of the strains isolated in this study could be further increased by optimizing the medium and cultural conditions.

*L. casei* f1, *L. paracasei* f2 and *L. paracasei* f3 could also degrade blending oils, peanut oil and sesame oil and they were separately tended to degrade blending oils, peanut oil and sesame oil. To identify the lipolytic peculiarity with the cell growth, *L. casei* f1, *L. paracasei* f2 and *L. paracasei* f3 were cultivated in the medium containing the corresponding preferred oils as the substrate. The degrading rate of the oil persistently increased from the late log phase to the stationary phase. The results were in accordance with the reports that the lipase production was generally released during the late logarithmic or stationary phase (Ghosh et al. 1996; Sharma et al. 2001). Among the various fatty acids inducing lipase secretion, oleic acid was found to be the best substrate for lipase production (Lakshmi et al. 1999). However, these three isolated strains all tended to hydrolyze the linoleic acid. Meanwhile, the amount of the fatty acids was obviously much higher in peanut oil and sesame oil than in blending oils, while the hydrolyze capacities of *L. casei* f1 and *L. paracasei* f2 to fatty acids were much stronger than that of *L. paracasei* f3. These results suggested that the degrading quantity and rate to the fatty acids were highly correlated with total weight of the corresponding fatty acids in the oil. Moreover, there were only a few lipases have been isolated which were specific for the position at which the majority of 18:2ω6 and 18:3ω3 fatty acids were situated in the triglyceride molecule, usually the central position (Brooksbank et al. 2007). However, these three isolated strains performed well in degrading linoleic acid, especially *L. paracasei* f3 with the degrading rate of 64.44%. These results were in line with the reported studies that lipolytic activity increased as the total amount of C18:n fatty acid esters in the vegetable oil increased (Lakshmi et al. 1999).

The lauric acid could be hydrolyzed by *L. casei* f1 which was slightly hydrolyzed by *L. paracasei* f2 and *L. paracasei* f3. The stearic acid could be hydrolyzed by *L. paracasei* f2 which was slightly hydrolyzed by *L. casei* f1 and *L. paracasei* f3. Prominently, only *L. paracasei* f3 could hydrolyze linolelaidic acid and linolenic acid (Fig. 5d). Although arachidonic acid has four double bonds like linolenic acid, it was barely degraded by these three isolated strains. It was probably because the presence of the double bonds was closer to the carboxyl groups in arachidonic acid making the esters resistant to attack by lipase.

Most of the microorganisms which could highly degrade fats, oils and greases were aerobic, such as bacteria from *Pseudomonas* spp, *Bacillus* spp, *Saccharomyces* spp. and so on (Ruiz et al. 2005; Paramithiotis et al. 2006; Hachemi et al. 2017; Bora et al. 2018). It was reported that *B. thermoleovorans* IHI-91 could utilize 93% olive oil during 7 h fermentation under the optimized condition (Markossian et al. 2000). The degradation rate was obviously higher than that of observed in this studied. To explain this, one speculation was that the *B. thermoleovorans* IHI-91 was aerobic and could obtain a large amount of biomass in a short time under the optimized conditions. The other assumption might be the olive oil was the best substrate for *B. thermoleovorans* IHI-91 while the most fitful substrate for the isolated strains in this work need to be explored. In addition, the aerobic microorganisms with high lipolytic activity were usually used to treat waste water and there was a bottleneck that the degrading rate reduced along with the air consumed (Brooksbank et al. 2007). Hence, the isolated strains in this work might be used together with those aerobic microorganisms for waste water treatment for the reason that the isolated strains were anaerobic and performed lipolytic activity to the oils commonly consumed in peoples’ daily life. Besides, the isolated strains could be used for the production of dairy related products owing to *L. casei* and *L. paracasei* are the usual starter in the dairy industry and their lipolytic activity might be enough for some dairy products, especially for the production of cheese which need a long time to ripe (Aravindan et al. 2007).

The probiotic properties of desirable bacteria are dependent on their capacity to resist to gastrointestinal acid and bile to colonize the surface of human intestinal cells and perform high anti-microbial activities to optimize the intestinal environment. Species of the genera *Bifidobacterium* and *Lactobacillus* are widely marketed for human consumption as probiotics in concentrated preparations available as capsules, powders, or liquid products, or else incorporated into milk-based functional foods (Perotti et al. 2014; Albadran et al. 2018). But the market of functional food industries is currently interested in new non-dairy foods which could have the potential to attract more consumers to functional products allowing also a more regular intake of probiotics. Therefore, the probiotic characterization of the *L. casei* f1, *L. paracasei* f2 and *L. paracasei* f3 were performed through standard in vitro experiments.

The first main barrier that the micro-organisms meet after ingestion is gastric juice, in which the inhibitory effect is strictly related to the low pH which could be buffered to pH 3.0 by the presence of the food (Pennacchia et al. 2008). In this study, pH 2.0 and pH 3.0 were used to identify the tolerance of the tested strains to the gastric juice. Furthermore, bile resistance is another important criterion in the selection of a culture as a dietary adjunct. Although the bile concentration of the GI tract varies, the mean intestinal bile concentration used for the screening of a resistant probiotic strain is usually 0.1% and 0.3% (Erkkila and Petaja 2000). *L. casei* f1, *L. paracasei* f2 and *L. paracasei* f3 demonstrated weak tolerance to the simulated conditions, while the viable counts under all conditions could still be higher than 10^6^ CFU/mL.

There might be some antimicrobial compounds in the supernatant because six indicator bacteria were inhibited by the supernatant of *L. casei* f1, *L. paracasei* f2 and *L. paracasei* f3. The antimicrobial capacity of LAB was due to the production of one or more active metabolites, such as organic acids, hydrogen peroxide, diacetyl, antifungal compounds, bacteriocins and bacteriocin-like inhibitory substances (Salomskiene et al. 2019). As the *L. casei* f1, *L. paracasei* f2 and *L. paracasei* f3 presented well antimicrobial characteristics, the corresponding compounds were worth to be studied. Meanwhile, the brilliant antimicrobial ability of the three isolated strains made them attractive to be applied in food industry.

## Supplementary information

**Additional file 1.** Additional tables.

## Data Availability

The data supporting the conclusions of this article are included within the article. Data and materials can also be requested from the corresponding author.
