# Supplementary material for: Isolation of novel Lactobacillus with lipolytic activity from the vinasse and their preliminary potential using as probiotics
Source: AMB Express. 2020 May 15;10:91. doi: 10.1186/s13568-020-01026-2 (PMC7229107; doi:10.1186/s13568-020-01026-2)
Supplement: Supplementary file 1 — Additional file 1. Additional tables. [file 13568_2020_1026_MOESM1_ESM.docx]

**Supplementary Tables**

Table S1 The morphological and growth-related characteristics of the bacteria in the enriching medium

| **Strain number** | **Sample** | **Morphological characteristics** |
| --- | --- | --- |
| 1 | S1 | Creamy, small and smooth colonies |
| 2 | S1 | Creamy, small and smooth colonies |
| 3 | S2 | Creamy, large and smooth colonies |
| 4 | S2 | Creamy, small and smooth colonies |
| 5 | S2 | Fungi like colonies |
| 6 | S2 | Fungi like colonies |
| 7 | S3 | Creamy, large and smooth colonies |
| 8 | S3 | Creamy, small and smooth colonies |
| 9 | S3 | Small and smooth colonies |
| 10 | S3 | Creamy, large and smooth colonies |
| 11 | S3 | Creamy, large and smooth colonies |
| 12 | S4 | Creamy, large and smooth colonies |
| 13 | S4 | Large and smooth colonies |
| 14 | S4 | Creamy, large and smooth colonies |
| 15 | S5 | Fungi like colonies |
| 16 | S5 | Creamy, small and smooth colonies |
| 17 | S5 | Fungi like colonies |
| 18 | S5 | Creamy, small and smooth colonies |
| 19 | S5 | Creamy, small and smooth colonies |
| 20 | S5 | Creamy, small and smooth colonies |

Table S2 The morphological characteristics and microscopic examination of the bacteria isolated from the screening medium

| **Strain number** | **Morphological characteristics** | **Microscopy inspection^1^** |
| --- | --- | --- |
| 1 | Creamy, large and smooth colonies | G^+^, rod shape |
| 2 | No colony | / |
| 3 | No colony | / |
| 4 | Creamy, large and smooth colonies | G^+^, rod shape |
| 7 | No colony | / |
| 8 | No colony | / |
| 9 | Creamy, small and smooth colonies | G^+^, rod shape |
| 10 | No colony | / |
| 11 | Creamy, small and smooth colonies | G^+^, rod shape |
| 12 | Creamy, small and smooth colonies | G^+^, rod shape |
| 13 | No colony | / |
| 14 | No colony | / |
| 16 | No colony | / |
| 18 | Creamy, small and smooth colonies | G^+^, rod shape |
| 19 | Creamy, small and smooth colonies | G^+^, rod shape |
| 20 | Creamy, small and smooth colonies | G^+^, rod shape |

^1^G^+^: Gram positive

Table S3 The growth and morphology characteristics of the bacteria isolated by the B-screening medium

| Strain number | Morphological characteristics |
| --- | --- |
| 1 | Creamy, small and smooth colonies |
| 4 | Creamy, small and smooth colonies |
| 9 | No colony |
| 11 | No colony |
| 12 | No colony |
| 18 | Creamy, small and smooth colonies |
| 19 | No colony |
| 20 | No colony |

Table S4 The 16S rDNA sequence of the isolated strains

| strain | 16S rDNA sequence |
| --- | --- |
| *L. casei* f1  *L. paracasei* f2  *L. paracasei* f3 | CCCCTGGCCGGCGTGCCCTATACATGCAAGTCGAACGAGTTCTCGTTGATGATCGGTGCTTGCACCGAGATTCAACATGGAACGAGTGGCGGACGGGTGAGTAACACGTGGGTAACCTGCCCTTAAGTGGGGGATAACATTTGGAAACAGATGCTAATACCGCATAGATCCAAGAACCGCATGGTTCTTGGCTGAAAGATGGCGTAAGCTATCGCTTTTGGATGGACCCGCGGCGTATTAGCTAGTTGGTGAGGTAATGGCTCACCAAGGCGATGATACGTAGCCGAACTGAGAGGTTGATCGGCCACATTGGGACTGAGACACGGCCCAAACTCCTACGGGAGGCAGCAGTAGGGAATCTTCCACAATGGACGCAAGTCTGATGGAGCAACGCCGCGTGAGTGAAGAAGGCTTTCGGGTCGTAAAACTCTGTTGTTGGAGAAGAATGGTCGGCAGAGTAACTGTTGTCGGCGTGACGGTATCCAACCAGAAAGCCACGGCTAACTACGTGCCAGCAGCCGCGGTAATACGTAGGTGGCAAGCGTTATCCGGATTTATTGGGCGTAAAGCGAGCGCAGGCGGTTTTTTAAGTCTGATGTGAAAGCCCTCGGCTTAACCGAGGAAGCGCATCGGAAACTGGGAAACTTGAGTGCAGAAGAGGACAGTGGAACTCCATGTGTAGCGGTGAAATGCGTAGATATATGGAAGAACACCAGTGGCGAAGGCGGCTGTCTGGTCTGTAACTGACGCTGAGGCTCGAAAGCATGGGTAGCGAACAGGATTAGATACCCTGGTAGTCCATGCCGTAAACGATGAATGCTAGGTGTTGGAGGGTTTCCGCCCTTCAGTGCCGCAGCTAACGCATTAAGCATTCCGCCTGGGGAGTACGACCGCAAGGTTGAAACTCAAAGGAATTGACGGGGGCCCGCACAAGCGGTGGAGCATGTGGTTTAATTCGAAGCAACGCGAAGAACCTTACCAGGTCTTGACATCTTTTGATCACCTGAGAGATCAGGTTTCCCCTTCGGGGGCAAAATGACAGGTGGTGCATGGTTGTCGTCAGCTCGTGTCGTGAGATGTTGGGTTAAGTCCCGCAACGAGCGCAACCCTTATGACTAGTTGCCAGCATTTAGTTGGGCACTCTAGTAAGACTGCCGGTGACAAACCGGAGGAAGGTGGGGATGACGTCAAATCATCATGCCCCTTATGACCTGGGCTACACACGTGCTACAATGGATGGTACAACGAGTTGCGAGACCGCGAGGTCAAGCTAATCTCTTAAAGCCATTCTCAGTTCGGACTGTAGGCTGCAACTCGCCTACACGAAGTCGGAATCGCTAGTAATCGCGGATCAGCACGCCGCGGTGAATACGTTCCCGGGCCTTGTACACACCGCCCGTCACACCATGAGAGTTTGTAACACCCGAAGCCGGTGGCGTAACCCTTTTAGGGAGCGAGCCGTCTAAGGTGACAAATG  GGCAGGGGCGGCGTGCCTATACATGCAAGTCGAACGAGTTCTTGTTGATGATCGGTGCTTGCACCAGAGATTCAACATGGAACGAGTGGCGGACGGGTGAGTAACACGTGGGTAACCTGCCCTTAAGTGGGGGATAACATTTGGAAACAGATGCTAATACCGCATAGATCCAAGAACCGCATGGTTCTTGGCTGAAAGATGGCGTAAGCTATCGCTTTTGGATGGACCCGCGGCGTATTAGCTAGTTGGTGAGGTAATGGCTCACCAAGGCGATGATACGTAGCCGAACTGAGAGGTTGATCGGCCACATTGGGACTGAGACACGGCCCAAACTCCTACGGGAGGCAGCAGTAGGGAATCTTCCACAATGGACGCAAGTCTGATGGAGCAACGCCGCGTGAGTGAAGAAGGCTTTCGGGTCGTAAAACTCTGTTGTTGGAGAAGAATGGTCGGCAGAGTAACTGTTGTCGGCGTGACGGTATCCAACCAGAAAGCCACGGCTAACTACGTGCCAGCAGCCGCGGTAATACGTAGGTGGCAAGCGTTATCCGGATTTATTGGGCGTAAAGCGAGCGCAGGCGGTTTTTTAAGTCTGATGTGAAAGCCCTCGGCTTAACCGAGGAAGCGCATCGGAAACTGGGAAACTTGAGTGCAGAAGAGGACAGTGGAACTCCATGTGTAGCGGTGAAATGCGTAGATATATGGAAGAACACCAGTGGCGAAGGCGGCTGTCTGGTCTGTAACTGACGCTGAGGCTCGAAAGCATGGGTAGCGAACAGGATTAGATACCCTGGTAGTCCATGCCGTAAACGATGAATGCTAGGTGTTGGAGGGTTTCCGCCCTTCAGTGCCGCAGCTAACGCATTAAGCATTCCGCCTGGGGAGTACGACCGCAAGGTTGAAACTCAAAGGAATTGACGGGGGCCCGCACAAGCGGTGGAGCATGTGGTTTAATTCGAAGCAACGCGAAGAACCTTACCAGGTCTTGACATCTTTTGATCACCTGAGAGATCAGGTTTCCCCTTCGGGGGCAAAATGACAGGTGGTGCATGGTTGTCGTCAGCTCGTGTCGTGAGATGTTGGGTTAAGTCCCGCAACGAGCGCAACCCTTATGACTAGTTGCCAGCATTTAGTTGGGCACTCTAGTAAGACTGCCGGTGACAAACCGGAGGAAGGTGGGGATGACGTCAAATCATCATGCCCCTTATGACCTGGGCTACACACGTGCTACAATGGATGGTACAACGAGTTGCGAGACCGCGAGGTCAAGCTAATCTCTTAAAGCCATTCTCAGTTCGGACTGTAGGCTGCAACTCGCCTACACGAAGTCGGAATCGCTAGTAATCGCGGATCAGCACGCCGCGGTGAATACGTTCCCGGGCCTTGTACACACCGCCCGTCACACCATGAGAGTTTGTAACACCCGAAGCCGGTGGCGTAACCCTTTTAGGGAGCGAGCCGTCTAAAGGGGGCCAAAGTTG  CCCTTGCGGCTGCCTATACATGCAAGTCGAACGAGTTCTCGTTGATGATCGGTGCTTGCACCGAGATTCAACATGGAACGAGTGGCGGACGGGTGAGTAACACGTGGGTAACCTGCCCTTAAGTGGGGGATAACATTTGGAAACAGATGCTAATACCGCATAGATCCAAGAACCGCATGGTTCTTGGCTGAAAGATGGCGTAAGCTATCGCTTTTGGATGGACCCGCGGCGTATTAGCTAGTTGGTGAGGTAATGGCTCACCAAGGCGATGATACGTAGCCGAACTGAGAGGTTGATCGGCCACATTGGGACTGAGACACGGCCCAAACTCCTACGGGAGGCAGCAGTAGGGAATCTTCCACAATGGACGCAAGTCTGATGGAGCAACGCCGCGTGAGTGAAGAAGGCTTTCGGGTCGTAAAACTCTGTTGTTGGAGAAGAATGGTCGGCAGAGTAACTGTTGCCGGCGTGACGGTATCCAACCAGAAAGCCACGGCTAACTACGTGCCAGCAGCCGCGGTAATACGTAGGTGGCAAGCGTTATCCGGATTTATTGGGCGTAAAGCGAGCGCAGGCGGTTTTTTAAGTCTGATGTGAAAGCCCTCGGCTTAACCGAGGAAGCGCATCGGAAACTGGGAAACTTGAGTGCAGAAGAGGACAGTGGAACTCCATGTGTAGCGGTGAAATGCGTAGATATATGGAAGAACACCAGTGGCGAAGGCGGCTGTCTGGTCTGTAACTGACGCTGAGGCTCGAAAGCATGGGTAGCGAACAGGATTAGATACCCTGGTAGTCCATGCCGTAAACGATGAATGCTAGGTGTTGGAGGGTTTCCGCCCTTCAGTGCCGCAGCTAACGCATTAAGCATTCCGCCTGGGGAGTACGACCGCAAGGTTGAAACTCAAAGGAATTGACGGGGGCCCGCACAAGCGGTGGAGCATGTGGTTTAATTCGAAGCAACGCGAAGAACCTTACCAGGTCTTGACATCTTTTGATCACCTGAGAGATCAGGTTTCCCCTTCGGGGGCAAAATGACAGGTGGTGCATGGTTGTCGTCAGCTCGTGTCGTGAGATGTTGGGTTAAGTCCCGCAACGAGCGCAACCCTTATGACTAGTTGCCAGCATTTAGTTGGGCACTCTAGTAAGACTGCCGGTGACAAACCGGAGGAAGGTGGGGATGACGTCAAATCATCATGCCCCTTATGACCTGGGCTACACACGTGCTACAATGGATGGTACAACGAGTTGCGAGACCGCGAGGTCAAGCTAATCTCTTAAAGCCATTCTCAGTTCGGACTGTAGGCTGCAACTCGCCTACACGAAGTCGGAATCGCTAGTAATCGCGGATCAGCACGCCGCGGTGAATACGTTCCCGGGCCTTGTACACACCGCCCGTCACACCATGAGAGTTTGTAACACCCGAAGCCGGTGGCGTAACCCTTTTAGGGAGCGAGCCGTCTAAGGTGGACAAAGTTTG |
